# Peptide Arrays for Kinome Analysis of Livestock Species

**DOI:** 10.3389/fvets.2014.00004

**Published:** 2014-10-14

**Authors:** Joanna Daigle, Brenden Van Wyk, Brett Trost, Erin Scruten, Ryan Arsenault, Anthony Kusalik, Philip John Griebel, Scott Napper

**Affiliations:** ^1^VIDO-InterVac, University of Saskatchewan, Saskatoon, SK, Canada; ^2^Department of Biochemistry, University of Saskatchewan, Saskatoon, SK, Canada; ^3^Department of Computer Science, University of Saskatchewan, Saskatoon, SK, Canada; ^4^United States Department of Agriculture, Agricultural Research Service, SPARC, College Station, TX, USA; ^5^School of Public Health, University of Saskatchewan, Saskatoon, SK, Canada

**Keywords:** peptide array, kinome, livestock, infectious disease, kinases

## Abstract

Reversible protein phosphorylation is a central mechanism for both the transfer of intracellular information and the initiation of cellular responses. Within human medicine, considerable emphasis is placed on understanding and controlling the enzymes (kinases) that are responsible for catalyzing these modifications. This is evident in the prominent use of kinase inhibitors as drugs as well as the trend to understand complex biology and identify biomarkers via characterizations of global kinase (kinome) activity. Despite the demonstrated value of focusing on kinome activity, the application of this perspective to livestock has been restricted by the absence of appropriate research tools. In this review, we discuss the development of software platforms that facilitate the development and application of species-specific peptide arrays for kinome analysis of livestock. Examples of the application of kinomic approaches to a number of priority species (cattle, pigs, and chickens) in a number of biological contexts (infections, biomarker discovery, and food quality) are presented as are emerging trends for kinome analysis of livestock.

## Introduction

Research into animal physiology and pathophysiology benefit the agriculture industry by enabling more effective breeding, management, and treatment of livestock. Ultimately, this is to the betterment of food safety, as well as animal health and productivity. As such, there is an impetus on the agricultural industry to remain vigilant about identifying and incorporating cutting-edge research technologies. In this regard, the agriculture industry has been largely successful in developing, recognizing, and integrating emerging technologies to considerable advantage. Cutting-edge genomic approaches, for example, have had a revolutionary impact on how we select, manage, and manipulate food crops (1, 2). Efforts to identify valuable genetic traits within livestock species indicate similar potential for the food animal industry (3, 4).

Beyond characterizing static genomic traits, molecular characterizations of dynamic responses can also provide insight into complex phenotypes (5, 6). Fortunately, genome sequencing and transcriptional profiling are based in experimental approaches that are relatively species-independent. In other words, once the genomic sequence of an organism is obtained, largely through species-independent protocols, subsequent steps for developing transcriptional profiling tools are well established, highly conserved, and species-independent. As such, there is a minimal lag for the implementation of cutting-edge genomic and transcriptional approaches by the livestock industry.

The strategy of defining cellular responses through characterizations of gene expression is not without caveats and concerns. In particular, the ability of transcriptional data to accurately reflect and predict cellular phenotypes has been called into question. Multiple levels of post-transcriptional regulation – gene silencing, mRNA stability, differential translational efficiencies, differences in protein turnover, compartmentalization of enzymes/substrates, and protein post-translational modification – all separate gene expression from cellular response, complicating the interpretation of transcriptional data (7). Given this, there is priority to define cellular responses at levels closer to the phenotype, often at the level of the proteome. This includes sub-disciplines of proteomics that seek to define cellular responses at the level of protein post-translation modifications.

The proteome is subjected to a range of deliberate post-translation modifications (8). These range from proteolytic processing to the enzymatic addition of various functional groups in order to regulate various aspects of protein structure and function. These modifying groups include, but are not limited to, glycosylation, alkylation, adenylation, and isoprenylation (8). The multitude of protein isoforms that result as a consequence of these modifications bestows considerable complexity onto the proteome and its characterization.

### Phosphorylation-mediated signal transduction

Phosphorylation, as catalyzed by protein kinases, is the predominant mechanism of post-translation control of proteins in eukaryotes. These reversible modifications often represent the defining event for induction of a cellular response and/or phenotype (9). Kinases tend to occupy central regulatory positions with involvement in the control of virtually every cellular behavior, from metabolism to cell cycle regulation to pathogen clearance (7). As such, defining cellular responses at the level of phosphorylation-mediated signal transduction has the potential to provide unobstructed insight and predictive power into phenotypes.

Kinases are intimately associated with many pathological states, providing further rationale for the value of defining responses at the level of the kinome (10). Kinases also represent excellent drug targets, giving researchers the opportunity not only to understand but also to influence, biology (11, 12). Second only to G-coupled receptors, the kinases are a highly targeted class of enzymes for drug development. In fact, many kinases inhibitors are already licensed as therapeutics, in particular for the treatment of cancer (13). While it is unlikely that kinase inhibitors will ever see extensive therapeutic use in livestock, emerging libraries of kinase inhibitors nevertheless represent an important resource for validation of experimental hypotheses.

There are clearly a number of distinct advantages associated with performing molecular characterizations at the level of phosphorylation-mediated signal transduction. Unfortunately, the ability to define dynamic patterns of protein phosphorylation within livestock has been hindered by the limited availability of required research tools (14). For example, the extent to which the phosphoproteome of a species can be monitored with phosphorylation-specific antibodies directly reflects the availability of these antibody reagents. The limited number of phosphorylation-specific antibodies that are commercially available for livestock has lead many livestock researchers to resort to antibodies that have been developed for other species, which can be problematic as the extent and specificity to which these antibodies cross-react with livestock proteins is often unverified.

Alternatively, it is also possible to characterize livestock phosphoproteomes through specialized mass spectrometry techniques (14, 15). A comparative examination of the relative merits of the various mass spectrometric approaches for phosphoproteome analysis has been reviewed elsewhere (14). A noteworthy feature of most of these approaches is that their costs and technical requirements can be quite prohibitive. Further, within these mass spectrometric approaches, differences in instruments and instrument settings can have strong influence on data outputs, complicating effective utilization of these techniques by non-experts.

### Peptide arrays for kinome analysis

An alternative for defining phosphorylation-mediated signal transduction activity is to focus on the activities of kinases rather than the extent of phosphorylation of the protein substrates (16). While there are obvious functional and conceptual linkages between the activity of a kinase and the extent of phosphorylation of its protein target, there are a number of experimental advantages to kinome analysis. The relative merits of kinomic and phosphoproteomic approaches have been discussed elsewhere (14).

The central prerequisite for development of a peptide array for kinome analysis is identification of kinase substrates that are suitable for array format. As the specificity of many kinases is determined by the positions flanking the phosphoacceptor site, it is possible to use short peptides as surrogate kinase substrates (17). For many kinases, the enzymatic characteristics (*V*_max_ and *K_m_*) for peptides representing their recognition sequences are very similar to that of the native protein substrate (18). Thus, it is possible to develop arrays for kinome analysis using peptides that represent priority phosphorylation events. The well-defined and highly conserved chemistry of kinase-mediated phosphoryl group transfer has proven well suited for arrays and array-based approaches are an effective platform for high-throughput, low-cost global surveys of cellular activity (7, 14).

**Figure 1:**
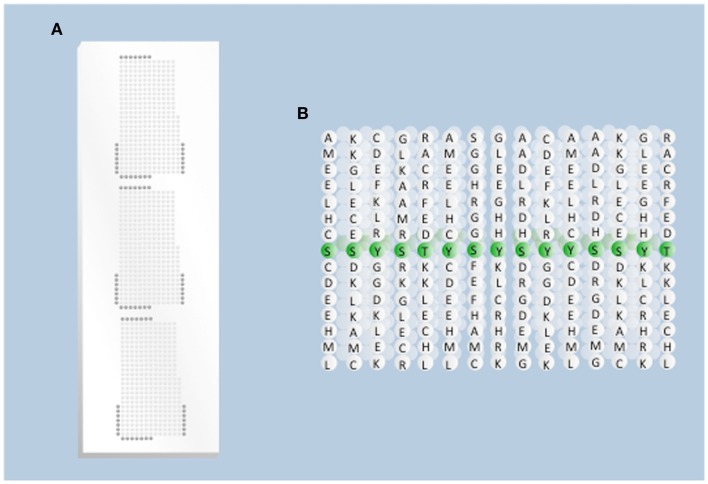
**Overview of array design**. **(A)** Physical presentation of the peptides. Each spot on the array represents a different peptide sequence presented within a grid of the total population of peptides (kinase substrates) to be considered. Each grid is replicated three times to generate technical replicates of each spot. Dark gray spots on the edge of the grid represent negative control peptides. **(B)** Peptides on the array. Each spot on the array represents a population of single peptides (typically 15 amino acids long) in which the central position is the phosphoacceptor residue.

For species that have been the focus of intensive research priority, such as humans and mice, detailed information relating to their phosphoproteomes is readily accessible within publically available databases such as PhosphoSite (19) or PhospoEML (20). From these databases, peptide sequences corresponding to priority phosphorylation events can be selected for incorporation onto an array (Figure 1). However, considerably less effort has been invested in defining the phosphoproteomes of other species, including livestock. The scarcity of information relating to the sequence contexts of phosphorylation events within livestock proteins is a critical obstacle to the development of peptide arrays. It is unlikely that the large-scale proteomic characterizations that are required to address this obstacle will be performed in the near future.

## Tools Enabling Kinome Analysis of Livestock

Novel bioinformatics tools have been developed to mitigate the difficulties associated with developing a peptide array for species with poorly defined phosphoproteomes. These approaches are based on the conservation of kinases and their targets across species. For example, 518 distinct human kinases have been identified and a similar number of bovine kinases, 512 have been predicted, each orthologous to a human kinase (8, 21). Interestingly, the degree of kinase conservation between human and bovine appears to be similar to that between human and mouse, which is commonly used as a model organism for human. Specifically, the mouse genome encodes 540 kinases, 510 of which are orthologs to human kinases (22). Similar conservation exists in the phosphorylation targets of the kinases and the subsequent biology that they regulate.

This alternative method for array development was demonstrated in 2009, when a bovine-specific peptide array was generated based on genomic information (23). Through this methodology, a library of peptide sequences representing predicted bovine phosphorylation sites was created using known human phosphorylation sites as queries for BLAST searches. When the query and its best match in the bovine proteome had few or no sequence differences, the match was considered a putative bovine phosphorylation site. The comparison of protein descriptions for the query and the hit sequences confirmed that the matches referred to orthologous proteins. These peptides were then printed onto arrays, creating a first-generation, bovine-specific array (23).

These bovine-specific peptide arrays were applied to define signaling events initiated in bovine monocytes following stimulation of various individual toll-like receptors (TLRs) (23). This kinomic investigation, whose results were validated through independent techniques, revealed a number of signaling events not previously been associated with TLR activation. This study also identified and validated receptor-specific signaling events that resulted as a consequence of distinct TLR-activations (23). That the preliminary application of the species-specific arrays was able to identify novel signaling events for this intensely investigated family of receptors highlighted the power of the kinomics approach and validated the potential for the development of species-specific arrays.

Another critical outcome of this investigation was quantitative insight into the extent to which phosphorylation sites and the immediately surrounding sequences are conserved across species (23). Of the nearly 900 peptides considered, nearly half were absolutely conserved between humans and cattle, a quarter showed a high level of sequence conservation – defined as one to 3 mismatches over the 15 residues – and the remainder shared no matches between these species. That phosphorylation sites between human and bovine orthologous proteins are not absolutely conserved indicates that a human kinome array would be of compromised value for analysis of bovine samples. Specifically, approximately 25% of the emerging data would have no biological meaning. Subsequent investigation of the extent of conservation of phosphorylation sites within the common livestock species highlights a similar trend and magnitude of conservation (Table 1). Of note is that the degree of phosphorylation site conservation between human and cow, pig, and sheep is comparable to the degree of conservation between human and mouse. Collectively, this was a landmark manuscript in demonstrating the potential, necessity, and mechanism for development of species-specific peptide arrays.

**Table 1:** **Phosphorylation site conservation between human, mouse, and various livestock species**.

Sequence differences	Mouse	Cow	Pig	Sheep	Honeybee	Chicken	Turkey
0	36.7	40.0	35.9	38.0	1.5	16.2	15.1
1	17.4	17.5	15.1	16.9	1.5	10.3	9.7
2	11.3	10.9	9.5	10.8	2.1	8.6	8.3
3	7.8	7.3	6.6	7.3	3.0	7.3	7.3
4	5.7	5.3	4.9	5.5	2.9	6.3	6.1
5	4.4	4.0	4.1	4.3	3.6	5.9	5.7
6	4.1	3.6	4.3	4.1	7.3	7.4	7.3
7+	12.5	11.4	19.5	13.2	78.1	38.0	40.4

*15-mer peptides having a central residue that is an experimentally determined phosphorylation site were downloaded from the PhosphoSitePlus database. The closest match to each peptide was identified in the proteome of each livestock species. The values in the first column represent the number of sequence differences between a human phosphorylation site and its best match in a given animal’s proteome, and the values in the remaining columns represent the percentage of sites having that number of sequence differences*.

### Predictive software tools (DAPPLE)

While effective, the methodologies employed for the development of the first species-specific kinome arrays were cumbersome and labor-intensive. The effort required to perform the cross-species analysis limited the number of phosphorylation sites and species that could be considered. As this methodology was limited to prediction of phosphorylation events based on those characterized for a single species, human, it also failed to capitalize on the sparse, yet important, phosphoproteome information of other species. While not a significant concern when generating arrays for mammalian species, this was of import for livestock species of greater evolutionary distance, such as poultry.

A software platform called DAPPLE was developed to address these limitations (24). The advantage of DAPPLE over the original approach was that it provided more definitive, objective criteria for identifying non-orthologous proteins. This increased confidence in the identities of the members of the predicted phosphoproteome and the ensuing array. DAPPLE was made freely available through a Web-based server^1^.

### Data analysis software tools (PIIKA and PIIKA2)

Originally, the kinome data generated via peptide arrays were processed through software platforms designed for analysis of microarray gene expression data. Superficially, this seemed a logical approach given the seeming similarities of the data: increased/decreased expression of a gene vs. increased/decreased phosphorylation of a peptide and both is being microarray-based techniques. As the kinome technology matured, however, it became apparent that these analysis platforms limited the extraction of biological information from kinomic datasets. Differences in the magnitude and chemical and biological nature of the data emerging from gene expression and kinome analysis rendered the statistical criteria typically used to assess gene expression data inappropriate for kinomic investigations (25).

To mitigate these limitations, a software platform called platform for intelligent, integrated kinome analysis (PIIKA) was developed specifically for the analysis of kinome data (25). PIIKA allows the user to identify peptides that are truly differentially phosphorylated between experimental conditions. A set of statistical criteria effectively addresses the naturally occurring technical and biological variability, allowing the user to quantify the magnitude and confidence of differential phosphorylation events. To facilitate the ease of data input and efficient extraction of results PIIKA was made freely available through a Web-based server^2^ with a graphical user interface designed for biological scientists who may be unfamiliar with command-line tools. The software is also available for local installation for research purposes through the same website.

While the establishment of PIIKA represented a critical step in the evolution of kinome analysis, the program was limited in terms of the sophistication of questions that could be asked of the data as well as the options available for data visualization. An updated version of the program, PIIKA2, offers improvements and advancements in the areas of cluster analysis, statistical outputs, and data visualization (26). PIIKA2 provides the option to perform statistical analysis to determine the extent to which groups of samples show similar kinome profiles and to identify subsets of peptides with consistent trends of modification across groups (26). Users are also better equipped to evaluate the biological significance of their kinome data through the calculation of false negative probabilities and positive and negative predictive values for *t*-tests between pairs of samples. Finally, the program also provides the option to visualize data via volcano plots, scatterplots, and interactive three-dimensional principal component analyses.

## Application of Peptide Arrays to Livestock

Species-specific peptide arrays for kinome analysis of livestock have been available for <5 years. In this short period of time, there have been extensive publications demonstrating the successful application on these arrays to a range of livestock species (cattle, pigs, chickens, and honeybees) and biological queries (infectious agents, metabolism, and biomarker discovery). This highlights the utility and versatility of the technology, both in terms of the range of species and the biological questions that can be addressed through the generation of species-specific and process-specialized arrays.

### Infectious disease

A priority application of livestock peptide arrays has been to characterize the signaling events that occur in response to infectious challenge. Understanding the molecular mechanisms of a host–pathogen interaction could inform strategies for rationale development of vaccines/therapeutics as well facilitate identification of biomarkers that anticipate disease susceptibility, severity, or outcome. Kinome analysis is particularly well suited for investigations of host–pathogen interactions as most host responses to infectious challenge are mediated through complex patterns of protein phosphorylation. From activation of the receptor, to transmission and amplification of the signal within the cell, to the ultimate activation of the biological response, there is a common dependence on kinase-mediated phosphorylation events (27). Further, many pathogens, in particular those that establish persistent infections, employ virulence mechanisms that involve subversion of host processes (28). Often these immune invasion strategies act via disruption of host signaling events, generally through kinase or phosphatase effector proteins (29, 30).

For pathogens that utilize kinases as pathogenic effectors, there is the opportunity to exploit the druggability of kinases in order to develop kinase inhibitor-based antimicrobials (31). Research on *Mycobacterium tuberculosis* indicates that treatment with imatinib, an FDA approved chemotherapeutic kinase inhibitor, facilitates bacterial clearance from a human fibroblast cell line (32).

#### Johne’s disease

Johne’s disease (JD), a chronic inflammatory disorder of the gastrointestinal tract of ruminants, is caused by *Mycobacterium avium* subsp. *paratuberculosis* (*MAP*) (33). There is a general consensus that the ability of *MAP* to subvert host immune responses represents a central obstacle to development of effective immunotherapeutics, indicating a deeper understanding of *MAP*’s virulence mechanisms is necessary for vaccine development.

Establishment of chronic infection by *MAP* depends on the bacteria’s ability to subvert host immune responses that can clear the infection. The necessity, and mechanisms, to overcome host immunity are well exemplified by the efforts of *MAP* to subvert bovine macrophages, thereby converting these central cellular effectors of host immunity into protected havens for survival, proliferation, and dissemination within the bovine host (34). A number of publications, to be discussed, have employed bovine-specific peptide arrays to understand the mechanisms by which MAP subverts various aspects of bovine macrophage function.

##### Toll-like receptor signaling

The innate immune system is an evolutionarily ancient system to recognize and clear pathogens independent of the adaptive immune system (27). Innate immune responses are triggered following recognition of pathogen associated molecular patterns by pattern recognition receptors, such as the TLRs (35). In response to the binding of receptor-specific ligands, TLRs induce a variety of innate immune responses.

Considerable evidence supports the importance of the TLRs to mycobacterial infections (36), including the potential to treat mycobacterial infections with TLR9 agonists (37). There is, however, controversy regarding the outcomes of mycobacterial engagement of any given TLRs, the ability for *MAP* to influence TLR-mediated innate immune responses and the value of TLR agonists as mycobacterial therapeutics. Bovine-specific peptide arrays were employed to evaluate the role of TLRs in JD, with emphasis on TLR9 due to the proposed use of TLR9 agonists as therapeutics for *MAP*.

**Figure 2:**
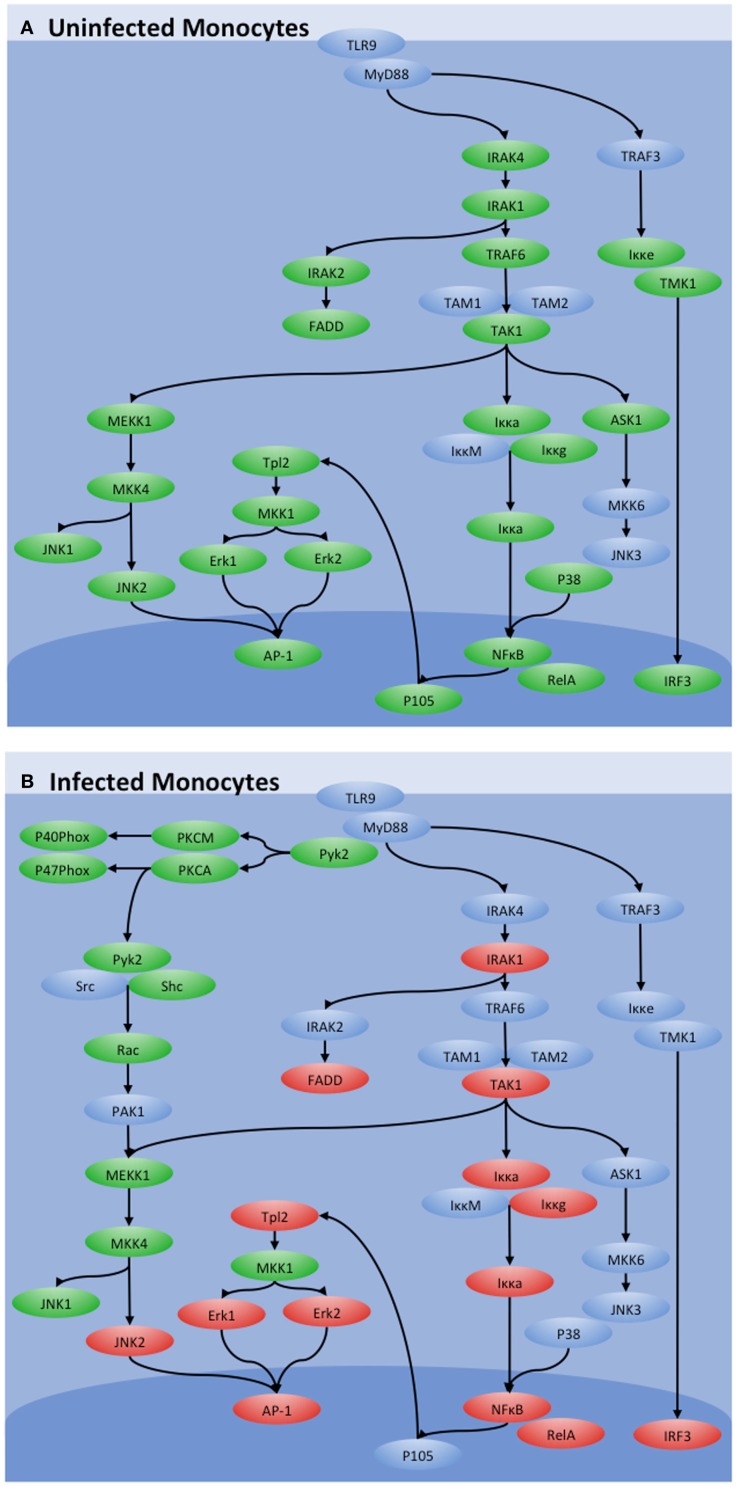
**Influence of *MAP* on TLR9 signaling**. TLR9 signaling in **(A)** uninfected and **(B)** *MAP*-infected bovine monocytes as determined by kinome analysis. Peptides with increased phosphorylation in a treatment are shown in red, those with decreased phosphorylation are shown in green, and those with no significant change are shown in blue. While canonical TLR9 signaling through IRAK4 and TRAF4 was observed in control monocytes, those pathways were shut down during *MAP* infection. TLR9 signaling was maintained, however, through an alternative pathway via Pyk2.

Infection of bovine monocytes with *MAP* resulted in a 10-fold increase in TLR9 expression, supportive of both the involvement of this receptor in promoting *MAP* clearance and the therapeutic potential of TLR9 agonists for JD (38). Critically, increased transcription of the TLR9 gene did not cause increased functional sensitivity to TLR9 agonists as stimulation of *MAP*-infected bovine monocytes with TLR9 agonists failed to induce cytokine responses previously associated with TLR signaling. Kinome analysis confirmed the absence of classic TLR-induced signaling in response to stimulation of the *MAP*-infected cells with TLR9 agonists. Instead, *MAP* redirects the classic TLR9 signaling through an alternate Pyk2-mediated route (Figure 2A). This appears to be functionally advantageous to the pathogen as treatment of *MAP*-infected bovine monocytes with Pyk2 inhibitors significantly reduced the intracellular load of *MAP* (Figure 2B) (38).

This investigation highlighted the importance of defining cellular responses at the level of the kinome. Specifically, the transcriptional data predicted increased sensitivity to TLR9 stimulation while kinome analysis offered a contrary perspective, demonstrating that TLR9-induced signaling was silenced in the *MAP*-infected cells, a conclusion that was validated by independent functional assays (38). This conclusion offers evidence and mechanistic explanation of why TLR9 agonists are unlikely to be effective as a treatment for JD. Further, by defining the signaling events that occur in *MAP*-infected monocytes, kinome analysis was able to suggest targets for therapeutic intervention that were experimentally validated.

##### Interferon gamma signaling

Interferon gamma (IFNγ) is a central effector of immune defense against intracellular pathogens, including *MAP* (39). Increased IFNγ is observed at the site of infection of cattle during the excretory, subclinical stage of JD (40), and increased production of IFNγ has been reported after stimulation of peripheral blood mononuclear cells (PBMCs) with *MAP* antigens (41). Increased production of IFNγ appears to represent an early response to MAP infection that continues throughout the persistent infection. This is not, however, effective in promoting MAP clearance as the pathogen appears to block the sensitivity of infected cells to this key cytokine; pre-treatment of macrophages with IFNγ promotes their ability to clear mycobacteria while the same treatment is ineffective when given post-infection (42, 43). Collectively, these results indicate that *MAP*-infected animals are able to produce, but not respond to, IFNγ.

**Figure 3:**
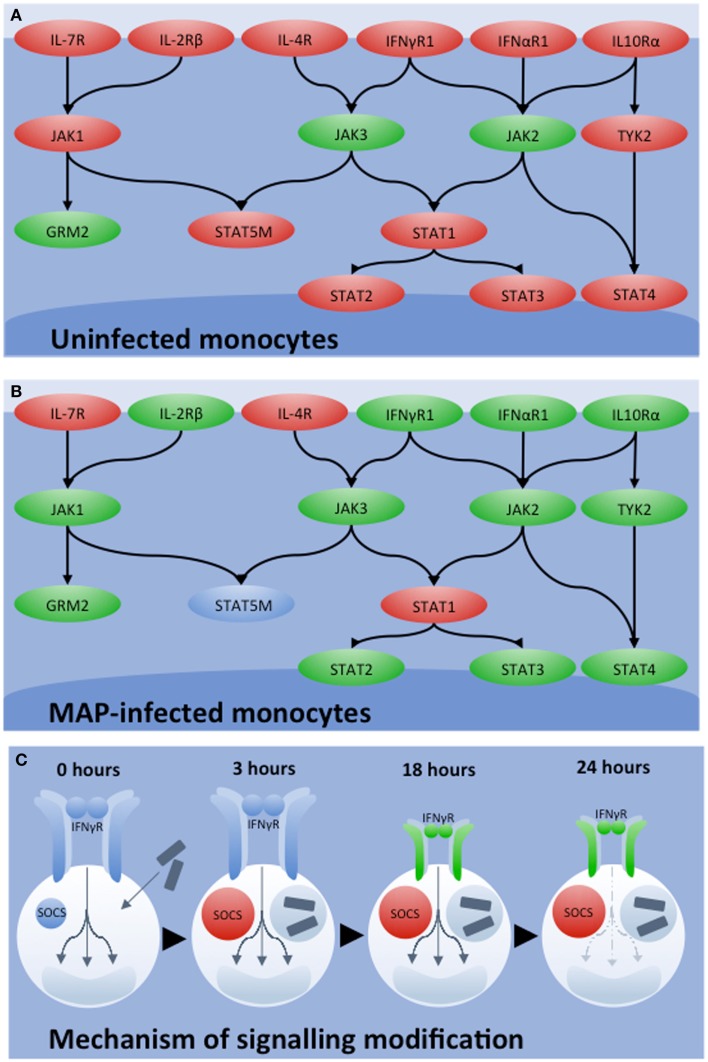
**Influence of *MAP* of IFNγ signaling**. JAK-STAT signaling networks characterized by kinome analysis in bovine monocytes under different conditions of **(A)** uninfected and **(B)** *MAP* infected. Relative degrees of phosphorylation are shown with genes showing increased phosphorylation in red, decreased phosphorylation in green, and insignificant change in blue. **(C)** Mechanisms of network silencing was determined by qRT-PCR. Shortly after *MAP* infection, SOCS1 and SOCS3 levels increased and remained so 18 h post-infection. While 3 h post-infection, no change in IFNγR1 and IFNγR2 expression were observed, both genes were down-regulated 18 h post-infection. These mechanisms together, explain the decrease in JAK-STAT signaling observed by kinome analysis 24 h post-infection.

Bovine-specific peptide arrays were utilized to characterize signaling responses initiated in bovine monocytes by IFNγ in the presence or absence of *MAP* infection (44). Stimulation of uninfected bovine monocytes with IFNγ resulted in activation of the JAK-STAT signaling pathway (Figure 3A). Conversely, activation of this classic response to IFNγ was not observed in *MAP*-infected bovine monocytes, rather there was strong evidence for repression of JAK-STAT signaling (Figure 3B). Subsequently, independent experiments verified that *MAP* infection decreased expression of the IFNγ receptor and increased expression of suppressor of cytokine signaling (SOCS)-1 and -3, which function as negative regulators of JAK-STAT signaling (Figure 3C). Accordingly, kinome analysis defined both the occurrence and mechanism of repression of IFNγ sensitivity (44). This information challenges the current dogma that the rational design of a protective vaccine for JD must include the induction of a T_H_1 or IFNγ immune-biased response.

##### Divergent responses in calf intestinal tissue

As many cattle that are exposed to *MAP* do not develop JD there is a priority to understand the mechanisms by which these animals resist infection. Insight on the nature of protective responses could inform strategies for developing protective vaccines or other therapeutics.

Kinome analysis was applied to calf intestinal tissue segments derived from an *in vivo* bovine intestinal segment model of JD in order to determine how *MAP* infection influences host responses at the site of invasion (45). The resulting kinome datasets clustered into two distinct groups, suggesting distinct, binary responses to *MAP*. Interestingly, two equally distinct *MAP*-specific immune responses, characterized by different antibody, T cell proliferation, and IFNγ responses, were observed (46). Most importantly, the kinomic groupings paired with the immune response groupings. This indicates arrays’ ability to discriminate complex cellular responses induced by *MAP* in the ileum and provides a novel method to understand mechanisms that alter the balance between cell-mediated and antibody responses to *MAP* infection (46). Furthermore, application of arrays to *in vivo* tissue samples represents a critical and ambitious step in using this technology to understand host–pathogen interactions.

#### Mycoplasma bovis

*Mycoplasma bovis* (*M. bovis*), a member of the bovine respiratory complex diseases (BRD), is responsible for a significant fraction of the economic losses associated with BRD in North America and Europe (47). *M. bovis* infections are associated with a number of disease states including pneumonia, mastitis, arthritis, and abortion (47). *M. bovis* uses respiratory epithelial cells as a port of entry with subsequent systemic dissemination through the bovine host via blood monocytes. Mechanisms employed by *M. bovis* to subvert host immunity and to achieve persistent cellular and systemic infection remain unclear.

Using bovine-specific peptide arrays in was demonstrated that *in vitro* infection of bovine monocytes with *M. bovis* influenced pathways relating to the caspase system, which would be anticipated to inhibit apoptosis (48). These observations were confirmed through functional assays, which verified that *M. bovis* infection delayed spontaneous apoptosis as well as apoptosis induced by either TNF-α or staurosporine. This may be a virulence mechanism used to prolong bacterial survival as well as facilitate infection dissemination. Kinome analysis also indicated that through regulation of the suppressor of cytokine signaling (SOCS) protein, *M. bovis* could influence the ability of the infected cells to produce and respond to critical cytokines involved in the control of infection (48).

#### Prion disease

Prion diseases are a novel paradigm of infection in which the infectious agent is the misfolded conformation (PrP^Sc^) of a normal self-protein (PrP^C^) (49). A number of prion diseases impact livestock animals including bovine spongiform encephalopathy (BSE) in cattle, scrapie in sheep, and chronic wasting disease (CWD) in cervids (deer and elk). Each of these diseases is associated with wasting, dementia, and, inevitably, death. There are many unanswered questions regarding the structural mechanisms of prion transmissibility as well as the pathological mechanisms of the misfolded species. Determining the biological role of PrP^C^, and the pathological mechanisms associated with PrP^Sc^, is a central priority to prion researchers.

There is strong evidence to suggest PrP^C^ is involved in signal transduction, however, the specific signaling events associated with PrP^C^ remain unknown (50, 51). There is similar uncertainty whether the cellular consequences of PrP^Sc^ formation reflect a loss, gain, or change of PrP^C^ signaling activity. Given the absence of clearly defined targets, the broad analysis made possible by kinome arrays appeared an ideal approach to define cellular mechanisms of healthy PrP^C^ and pathogenic mechanisms of prion disease.

While a definitive biological ligand for PrP^C^ had yet to be identified, it had been demonstrated that neuronal signaling can be activated by antibody-induced dimerization of PrP^C^ (52). Conversely, treatment of neurons with a specific PrP peptide fragment (PrP 106–126) activates prion disease-like responses that are thought to reflect PrP^Sc^ signaling (53). To determine the signaling events associated with PrP^C^, as well as to characterize the changes in signaling patterns with conversion to PrP^Sc^, kinome analysis was performed on healthy neuronal cells following treatment with either dimer-inducing antibodies (PrP^C^ signaling) or pathogenic peptide (PrP^Sc^ signaling).

Kinome analysis revealed unique patterns of signal transduction in response to each treatment (54). Specifically, antibody-induced dimerization initiated mitogen activated protein kinase (MAPK) signaling, whereas the activation of neurons with the “pathological peptide” activated vascular endothelial growth factor (VEGF) and phosphoinositide-3 kinase (PI3K) signaling pathways. These conclusions were confirmed through independent approaches that included phosphorylation-specific antibodies as well as functional assays (54).

The activation of distinct patterns of signaling within the same cell type following distinct methods of PrP activation was significant for demonstrating the functional versatility of PrP as a signal transduction molecule. It also highlighted signaling events that may be unique to the pathological condition and, therefore, logical targets for therapeutic intervention.

### Metabolism

There is widespread appreciation that intestinal microflora influence the health, growth, and productivity of livestock animals (55). Typically, this is considered from the perspective of how probiotics or food additives benefit livestock health and productivity. From a food safety perspective, appreciation also exists that certain commensal bacteria of livestock represent zoonotic threats to humans. Emerging evidence suggests that such bacteria, despite not causing disease symptoms or pathologies in livestock, can nevertheless have systematic consequences to the animal that influence growth, health, and product quality. For example, *Salmonella enterica* serovar Typhimurium (*Salmonella* Typhimurium) is a human pathogen responsible for a considerable portion of food borne illnesses (56). Despite its pathogenicity in humans, it has long been considered a chicken commensal as the bacterium colonizes and persists in the cecum of older chickens without any distinct pathology. Kinome analysis using a chicken-specific peptide array was applied to investigate the hypothesis that this bacterium has important systemic effects to the host, including changes to metabolic pathways of skeletal muscle.

Through the development and application of a chicken-specific peptide array important metabolic changes were identified in the skeletal muscle of *Salmonella* Typhimurium infected chickens (57). These changes affected fatty acid and glucose metabolism through the 5′-adenosine monophosphate-activated protein kinase (AMPK) and the insulin/mammalian target of rapamycin (mTOR) signaling pathway. As these pathways have an explicit influence on fatty acid and glucose metabolism, these findings could have implications for animal health and production. These data also suggest that it may be appropriate to redefine perspectives on how the presence of *Salmonella* spp. in the intestine of chickens can have systemic effects on host metabolism in the absence of clinical disease (57). The successful development of a species-specific peptide array for a non-mammalian livestock species is also significant and strongly suggests that similar arrays are possible for other poultry.

### Biomarker discovery

Conceptually, the simplest biomarkers are those associated with changes in the sequence of a nucleic acid, protein, or both. More complex single-molecule biomarkers may be associated with more discrete characteristics, such as patterns of expression or localization. Biomarkers for which there is a simple and direct relationship between a molecular characteristic and a phenotype are conceptually and mechanistically attractive; however, this likely underestimates the complexity of many phenotypes. It may be necessary to look beyond a “single gene, single phenotype” paradigm and to adopt a more global perspective; a perspective enabling consideration of the interplay between complex networks of biomolecules. Understanding functionally complex phenotypes, whether at transcriptomic, proteomic, or kinomic levels, depends on research tools that effectively monitor systems level changes within appropriate samples. As the tools become available to perform kinome investigations of livestock, as well as to probe the emerging data for phosphorylation patterns that predict outcomes, there is opportunity to identify and apply complex kinomic biomarkers.

#### Kinotypes

One of the challenges in working with outbred species such as humans or livestock is that there is often considerable variability in individual responses to a given stimulus. This diversity of responses is likely governed by interplay between a combination of genetic, epigenetic, environmental, and situational factors. Such variability is commonly observed in the responses of livestock to a diverse range of stimuli including infectious challenge, vaccines, drugs, and immunotherapeutics. As such, it was reassuring that early kinomic investigations of livestock revealed unique, animal-specific patterns of baseline kinome activity (38, 44, 46). While the kinomic baselines for the individual animals were often distinct, there was nevertheless conserved, though not identical, responses to a treatment condition or stimulus. This indicates that phenotypic differences reflect, and may even result from, unique cellular kinome environments. The intimate relationship between phenotype and kinotype supports the hypothesis that differences in global signaling patterns can be used as biomarkers.

As a first step to determine the extent to which kinome profiles differ among individuals and over time, peptide arrays were applied to define signaling profiles of PBMCs isolated from humans and pigs in a temporal fashion, samples were isolated from each individual once a week for a 1 month period. Kinome analysis was performed utilizing a chimeric pig/human array. These pig/human arrays were designed to only include peptides that are absolutely conserved in sequence for pig and human phosphorylation events. As such, this array is equally valid for either species and eliminates potential technical issues that might relate to comparison of kinome data from different arrays (58). The rationale and mechanisms for development of these species-chimeric arrays are discussed in greater detail later in the review.

**Figure 4:**
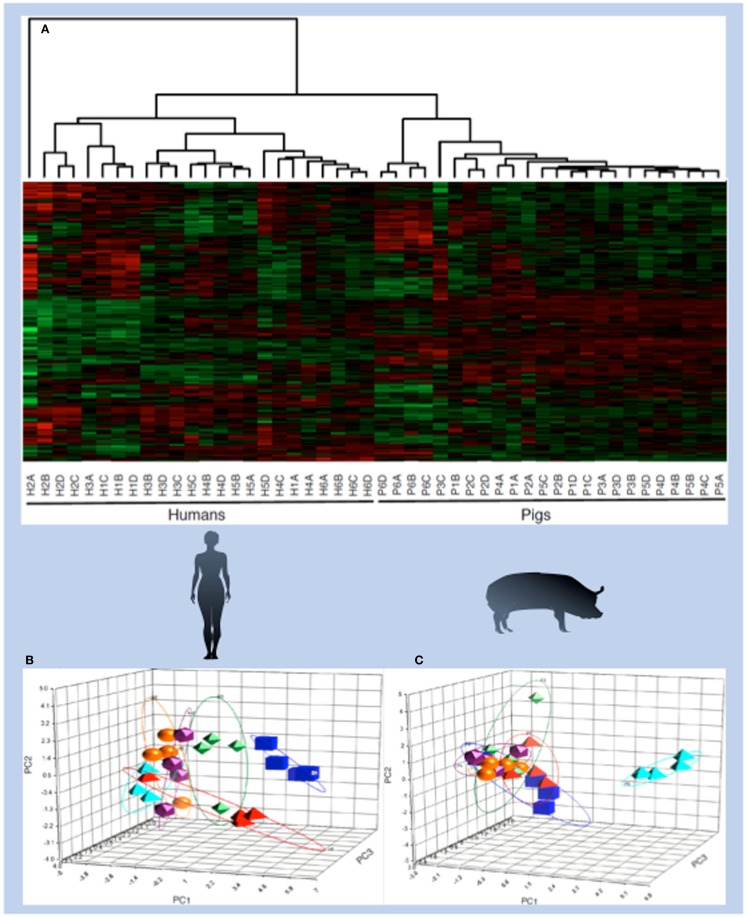
**Species- and individual-specific kinotypes**. PBMCs were isolated once each week for four consecutive weeks from six human individuals that were diverse in age, gender, diet, and health characteristics. PBMCs were also isolated from six pigs, which were littermates fed the same diet and housed in the same environment, according to the same schedule. Cell extracts from each sample were subjected to kinome microarray analysis. The resulting kinome profiles were analyzed via hierarchical clustering, in which each column represents a single individual at a given time point. Labels indicate human (H) or pig (P), the individual number (1–6), and the time point [**(A)** for the first week, **(B)** for the second week, and so on). (1-Pearson correlation) was used as the distance metric, while McQuitty linkage was used as the linkage method. Colors indicate the average normalized phosphorylation level of nine replicates of a single peptide, with red indicating increased phosphorylation and green indicating decreased phosphorylation. Color intensity indicates the magnitude of phosphorylation change. Clustering occurred across species lines and also between individuals, as shown by PCA analysis of human **(B)** and pig **(C)** kinome profiles. The principal components in the human PCA plot do not necessarily correspond to the same variables as those in the pig PCA plot.

Within the human and porcine kinome datasets there was clear evidence for species-specific kinome profiles; datasets relating to porcine samples were consistently distinct from human samples (58). Furthermore, within each species-specific grouping, individual-specific clustering was evident, indicating that each subject had a distinct and conserved pattern of signaling (Figure 4). This outcome was anticipated for the human subjects, who were of variable age, gender, and health, but was surprising for the porcine subjects who were littermates (brothers and sisters), maintained in the same environment and fed the same ration (58).

The ability of the arrays to reliably detect signaling differences within the porcine subjects highlighted the power of the kinome approach. The arrays were able to quantify cellular differences among individual subjects that likely reflect minor genetic and epigenetic factors, as well as interplays between complex mixtures of biomolecules. Considering the use of large animals as models of human disease, there will likely be a number of implications of the existence of species-specific kinotypes for the selection of animal models and the interpretation of results. Within the livestock industry, kinome biomarkers may be used to guide diagnosis and treatment, as well as for selecting commercially valuable phenotypes.

#### Honeybees and colony collapse disorder

While not within the traditional realm of livestock, honeybees are a vital component of the agriculture industry. Nearly a third of the world’s food crops depend upon pollination by honeybees and they are important food producers in their own right. The recent global collapse of honeybee populations is cause for considerable concern and has prompted calls for research tools that offer insight into the mechanisms of colony collapse disorder and identify biomarkers for the breeding of resistant bees (59, 60).

**Figure 5:**
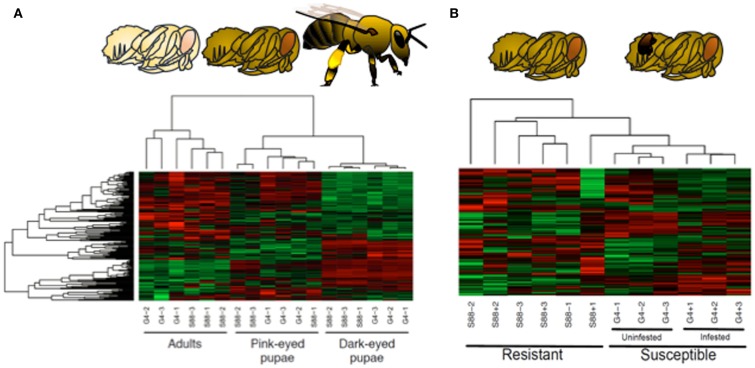
**Kinome analysis reveals developmental and phenotypic-specific signaling profiles in honeybees**. **(A)** Developmental stages. Honeybee developmental stages include pink-eyed pupa, dark-eyed pupa, and adult. Kinome arrays were able to discriminate effectively between these developmental stages as shown in hierarchical cluster analysis in which colors indicate normalized phosphorylation intensity with green indicating decreased phosphorylation and red indicating increased phosphorylation for the peptide’s average intensity over the nine replicates. Color intensity shows the level phosphorylation change. (1-Pearson) correlation was used to determine distance while McQuitty was used to determine linkage. One bee is represented by each column (nine bees total, three per treatment). **(B)** Susceptibility phenotypes and *Varroa* mite infection status. Clustering of dark-eyed pupae also correlated with *Varroa* mite susceptibility and infection status. Susceptible bees were also treated with *Varroa* mites or uninfected.

To address this challenge, a bee-specific peptide array was developed and applied to characterize honeybee families of distinct susceptibilities to *Varroa* mite infection, a primary suspect as a causative of colony collapse disorder (61). Kinome analysis was performed on whole bee extracts of each phenotype at three stages of development: pink-eyed pupae, dark-eyed pupae, and adults. The emerging kinome profiles offered overwhelming support for both developmental and phenotype-associated patterns of kinase activity (62). Firstly, each developmental stage is associated with a distinct signaling profile, within these groupings, sub-groupings corresponding to the different susceptibility phenotypes were observed (Figure 5).

The presence of kinome profiles that reflect, and presumably predict, a critical phenotype suggests that the potential to use these differences in signal patterns as biomarkers to guide breeding efforts. Within this work, it was determined that an array of as few as five strategically selected peptides could reliably (*p* < 0.05) discriminate the two phenotypes (62). Through the identification of the peptides with the greatest potential to discriminate the phenotypes, it may be possible to develop a focused, minimal array of value for breeding programs as well as for verification and assurance of *Varroa* mite resistance or other phenotypes for sales and marketing.

There were a number of important outcomes of this investigation. Firstly, the generation of a peptide array for a species as evolutionarily distant from mammals as honeybees offered confidence of the ability of DAPPLE to accurately predict the phosphoproteome of any species. Secondly, the successful application of kinomic approaches to whole organism extracts demonstrated the ability to apply the technology to samples of considerable biological complexity. Finally, there was a significant correlation between the phenotypes and kinotypes of these bees, which validated the hypothesis that kinome analysis is an appropriate level to identify biomarkers of complex phenotypes.

## Emerging Trends

### Complexity of samples

Initial kinomic investigations sought to minimize biological complexity by restricting investigations to either cell lines (54) or highly purified primary cells, such as monocytes (38, 44). More recent efforts have evolved toward samples of greater biological complexity that more accurately reflect the *in vivo* environment. This includes investigations of mixed cell populations, including PBMCs (58), tissue biopsies (57), and intestinal wall segments (46). Most recently, in the case of honeybees, peptide arrays have been successfully applied at a whole organism level (62). The arrays extracted meaningful biology from these complex samples, providing confidence that the technology is sufficiently robust and sensitive for its application to clinical samples and scenarios – moving it away from test tube investigations and toward the animal.

### Sophistication of questions

Initially, kinome analysis experiments were structured with priority on identifying peptides that were differentially phosphorylated in a treatment relative to a control. Functional linkages between these differentially phosphorylated peptides were then utilized to identify pathways involved in the response to the stimulus. While this remains a critical foundation of kinome analysis, there is also appreciation that this approach does not realize the full potential of information available within kinomic datasets.

Through PIIKA 2, it is possible to define more discrete features of the kinomic data. For example, within an experiment, it is often possible to define sub-groupings based on *a priori* knowledge of biological differences or phenotypes. It is then possible to interrogate the kinomic data to identify patterns of phosphorylation that correspond to these forced groupings. Through identification of signaling patterns that cluster with defining grouping characteristics investigators are able to evaluate the cellular basis of the phenotype as well as to identify biomarkers associated with this trait. For example, consider a scenario in which samples are taken from cattle with a genetic propensity to a particular disease. These animals can then be divided into two groups corresponding to those that contract the disease and those that do not. Through the identification of a subset of peptides that have similar responses in animals of the same group and different responses across groups, potential biomarkers for this disease will be discovered. The opposite situation could be considered as well, that is, a group of putative biomarker could be validated through these sub-groupings. Any biomarkers that do not correspond to these grouping would thus be identified as poor biomarkers for a given condition.

### Chimeric arrays

Many phosphorylation events, and the biological responses that they regulate, are reasonably well conserved across species. If for a particular phosphorylation event the amino acid sequence surrounding the phosphoacceptor site is absolutely conserved across two species, a peptide representing this sequence is equally applicable for kinome analysis of either species. As such, when selecting peptides for an array, it is possible to include the specific criteria of conservation across priority species such that the resulting array will be equally appropriate for either species. This adds value by expanding the potential range of application of a particular array and provides a consistent tool for evaluating cross-species responses, which may be of significance in strategic selection of appropriate animals to serve as models of disease as well as interpretation of the emerging results.

### Species range

Arrays have already been developed and validated for a number of species of priority to the livestock industry, including cattle, pigs, and chickens. Arrays for other priority food animals, such as sheep and turkeys, have also been generated and are being employed. Similar efforts are underway to develop arrays for a number of companion animals, including dogs, cats, and horses. Given the relative ease species-specific array development, the list of animals characterized through species-specific kinome arrays will continue to expand.

For food animal species, the overall priority is typically to ensure the health of a population of animal rather than an individual animal. This strongly influences the way in which kinome, and other scientific data, is applied. For example, the costs of treatments that are provided to food animals must be consistent with the commercial value of the products emerging for that animal. For this reason, the treatment of food animals with kinase inhibitors is not likely to represent a practical, cost-effective approach. There are also concerns about the safety, and perception of safety, of animals’ treatment, that will generate products for human consumption.

In contrast, the treatment options of companion animals is driven more by emotional rather than economic considerations. Indeed, kinase inhibitors are already being employed as chemotherapeutics for dogs, cats, and horses. For these animals, the peptides arrays might provide valuable information to informing treatment strategies and monitoring therapeutic outcomes. Further, understanding the cellular mechanisms of a disease may also provide insight into diagnosis, progression, and pathology.

While this is in and of itself a noble and worthwhile pursuit, there is also the larger context that cancers within these species may provide effective models and learning opportunities to better understand and treat human cancers (63). In addition, within agricultural species the regulatory and experimental barriers to the use of kinase inhibitors as therapeutics or phenotypic modulators is significantly less than in humans.

## Conclusion

Maintaining animal health and productivity is a central priority to the livestock industry. Increasingly, this end is achieved via the application of cutting-edge research technologies that consider and characterize animals at a global, molecular level. This perspective lends itself to a more comprehensive understanding of cellular events that are associated with processes and phenotypes of priority to the livestock industry. Peptide arrays for kinome analysis represent an emerging technology with great potential to contribute to the molecular perspective on animal health.

From the examples discussed within this review, there is a clear priority for the application of the arrays within the context of host–pathogen interactions. They demonstrate considerable capacity to further our understanding of the pathogenic mechanisms of a variety of livestock infectious agents. This is of considerable import as the complex immune evasion strategies employed by these pathogens are often barriers to the development of effective vaccines and therapeutics. Ultimately, the value of the arrays in this capacity will be determined through an evaluation of the contributions of this information to the development of effective vaccines and/or therapeutics.

An equally important, but slightly less daunting, challenge for the arrays will be biomarker discovery. As evidence of the potential to employ phosphorylation-associated biomarkers as indicators of important phenotypes grows, there is likely to be interest by the livestock industry to identify such biomarkers for priority species. For honeybees, strong evidence already demonstrates the potential to use differential kinases’ activity to predict important phenotypic traits. The evidence of temporally stable kinome fingerprints, or kinotypes, lends support to the overall concept that phenotypic differences may be reflected at the level of the kinome.

Kinome peptide arrays may also be utilized to assess food quality, as evidenced by a number of recent phosphoproteomic studies of food quality. Outcomes of these investigations include the identification of phosphoproteins that were identified as biomarkers for beef tenderness (64). Analysis of pork also suggested that protein phosphorylation levels could be biomarkers for metabolic processes that dictate meat quality (64). Additionally, proteome analyses in fish species suggested that changes in several metabolic and matrix proteins, including phosphoproteins, were indicative of flesh quality (64). The application of kinome analysis to food animals is not limited to meat quality, as comparison of milk samples collected before and after infection revealed differential phosphorylation of casein. The change in casein phosphorylation post-infection lead the authors to suggest that this change following infection could be used as a marker for infection, and further, a marker for milk quality (65).

**Figure 6:**
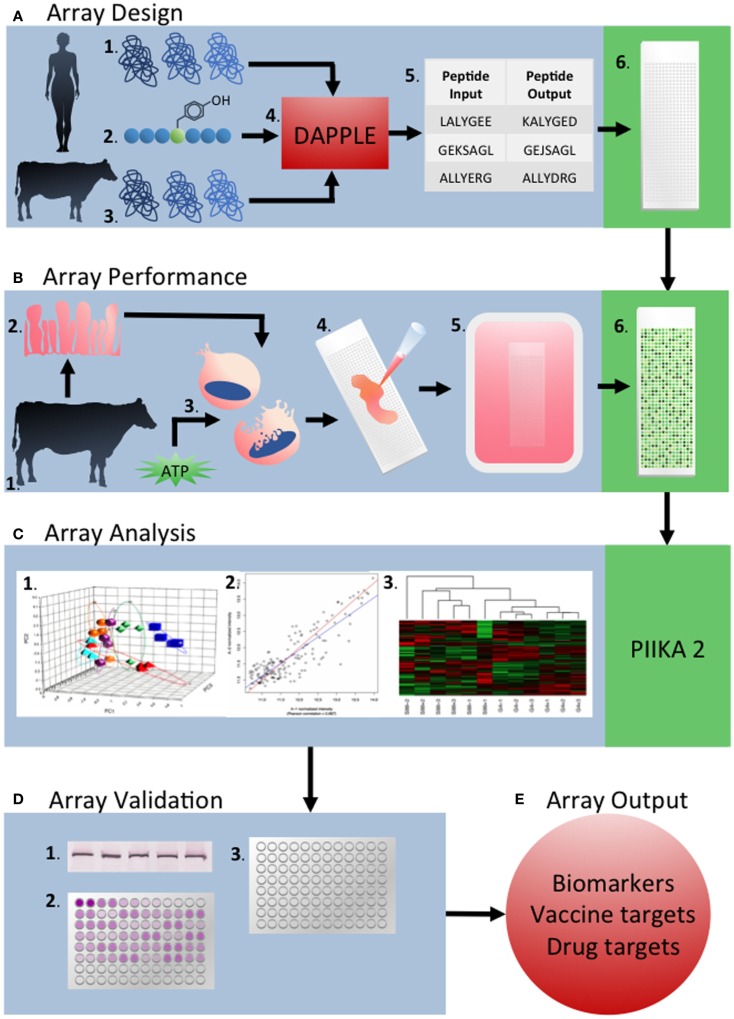
**Overview of the development and application of peptide arrays for kinome analysis**. **(A)** Array design: the human proteome (1) and known phosphorylation target sequences from previous phosphoproteomic studies (2), as well as the target species’ proteome are used as inputs into DAPPLE (4). This creates updated phosphorylated peptide sequences taking into account sequence differences between species (5). Peptides are chosen and spotted onto the array nine times each. **(B)** Array performance: sample tissue, cells, or cell line from the target organism (1) are collected (2). These samples are lysed and activated with ATP (3) before application to the array (4). After incubation, arrays are stained with Diamond ProQ phosphospecific stain (5) **(C)** Array analysis: raw fluorescence data is uploaded into PIIKA 2 and analyzed by a variety of statistical measures including chi-squared test, *t*-test, *f*-test, and others. PCA analysis (1), gene grouping (2), and heat MAPs (3) are also created by PIIKA 2. **(D)** Array validation: the results of a given array are validated using appropriate techniques to validate the results. Examples of validation experiments include western blotting (1) qRT-PCR (2), and ELISA (3). **(E)** Array outputs: array outputs include biomarkers, vaccine targets, drug targets, and targets for other therapeutics.

From these collective examples, we believe there is strong evidence for the value of kinome analysis of livestock species. Marked improvements have been made in the design and performance of kinome experiments over the last 5 years, including the establishment of a defined pipeline for the development of species-specific arrays, as well as analysis and interpretation of the emerging data to facilitate the extraction of important biological information (Figure 6). Further developments in array technology and kinome data analysis will only accelerate the application of this technology to a broader range of species and biological questions.

## Conflict of Interest Statement

The authors declare that the research was conducted in the absence of any commercial or financial relationships that could be construed as a potential conflict of interest.

## Footnotes

^1^http://saphire.usask.ca/saphire/dapple/

^2^http://saphire.usask.ca/saphire/piika/
